# North-Western Medical Society

**Published:** 1853-06

**Authors:** 


*North-Western Medical Society.*

Agreeable to a call, signed by the physicians of the City of Dubuque, a Meeting was held at the office of Dr. Kirkup in this city on the 4th of November, 1852, preparatory to the organization of a MEDICAL SOCIETY.

Dr. Sprague of Dubuque was called to the chair, and Dr. E. Kirkup appointed Secretary.

A resolution, declaratory of the object of the meeting was adopted, and on motion a Committee of three was appointed by the chair, to draft a constitution and by-laws for the government of said proposed Medical Society.

The following persons were appointed said Committee: Drs G. W. Richards, Asa Horr, and F. C. Smith.

On motion, the meeting adjourned for one week.

Nov. 11.

Assembled, agreeable to adjourment, and in the absence of Dr. Sprague, Dr. J. W. Finley, was called to the chair.

The Committee, appointed to draft a constitution and By Laws, submitted their Report, which on motion was accepted and the Committee discharged.

The Report was, on motion, taken up article by article, read, discussed, and amended, so as to meet the views of a majority present, and then laid upon the table, to be reported to a more general meeting, voted to be held at Dubuque on the second Tuesday of January, 1853.

On motion, the Secretary was authorized to procure printed circulars to be sent to the physicians of this and the surrounding counties, apprizing them of the time and objects of said adjourned meeting, and inviting their co-operation.

On motion, adjourned to meet as above specified.

Tuesday, Jan. 11, 1853.

The meeting assembled at the City Hotel, at the time specified, and was organized by choosing Dr G. W. Richards, of Dubuque, chairman, and Dr. F. Coleman Smith, Secretary.

The proceedings of the preliminary meetings were read, and on motion, the Constitution and By-Laws referred to in said proceedings, were taken up for the action of this meeting.

Some slight amendments were made, when, on motion, they were unanimously adopted, and are in the following words, to wit:

(APPENDED.)

After the adoption of the Constitution and By-Laws, the meeting resolved itself into a Committee of the whole, for the examination of diplomas, Licences, &c., and reported the following persons as possessing the requisite testimonials, each of whom came forward and subscribed to the Constitution:

| | |
|---|---|
| G. W. Richards, of Dubuque. | Harrison Holt, of Dubuque. |
| R. S. Lewis, " " | F. Coleman Smith, " |
| Asa Horr, " " | John W. Finlay, " |
| Thos. Scott, " " | W. R. McMahan, " |
| Robt. I. Thomas " " | A. E. Smith, Delaware Co. |

John F. Ely, Linn County.

The Meeting then proceeded to the permanent organization of the NORTH-WESTERN MEDICAL SOCIETY, by the Election of the following Officers for the ensuing year viz:

*President..*

G. W. RICHARDS, Dubuque.

| *1st. V. President.* | *2d. V. President.* |
|---|---|
| J. E. ELY, Linn Co. | HARRISON HOLT, Dubuque. |

| *Corresponding Sec.* | *Recording Sec.* |
|---|---|
| ASA HORR, DUBUQUE, | F. COLMAN, SMITH. |

*Treasurer.*

R. S. LEWIS, Dubuque.

*Censors.*

G. W. RICHARDS, ASA HORR, J. F. ELY.

After a brief but appropriate address from the President, the Society adjourned to meet at the office of Dr. Smith, to-morrow morning at 9 o'clock.

Wednesday Morning, Jan. 12.

The Society met according to adjournment, and was called to order by the President.

On motion, resolved, that when we adjourn it be to meet in this city, on the first Tuesday, of June next, at 3 o'clock P. M.

The President announced the following persons, as appointed to read Essays before the Society at its next meeting, viz:—Dr. John F. Ely, of Cedar Rapids, and Dr. G. D. Wilbur, Mineral Point, Wis.

On motion, a Committee was raised to report at the Annual Meeting, on the 1st Tuesday of Jan. next, on the Medical Topography of this section of country. The Committee consists of Drs. Asa Horr and Harrison Holt, of Dubuque, and Dr. A. E Smith, of Delaware County.

On motion, a Committee was raised to report at the meeting in June next, on Endemic Diseases. The following persons compose this committee.—Drs. Richards, of Dubuque; Newhall, of Galena; Van Dusen, of Mineral Point; Andros, of Garnavillo; Smith, of Hopkinton; and Ely, of Cedar Rapids.

On motion of Dr. Horr, the Code of Medical Ethics, of the National Medical Society, was adopted as the Code of this Society.

(APPENDED.)

On motion, Dr. Harrison Holt, of Dubuque, was appointed Delegate to the National Medical Society, to be holden at New York city on the 3d day of May next.

On motion, a Committee was appointed to report the form of a Seal and Diploma for the Society. The President appointed Drs. Lewis, Finlay and Thomas, said Committee.

On motion, ordered, that the proceedings of the meeting, together with the Constitution and By-Laws, and the Code of Ethics, be published in pamphlet form, and that a synopsis of the same be furnished to the publishers of the Western Medico-Chirurgical Journal at Keokuk, and the North-Western Medical Journal, at Chicago, with a request that they publish the same.

Adjourned to meet at Dubuque, on the first Tuesday, of June next.

G. W. RICHARDS, President.

F. COLEMAN SMITH, Rec. Sec.

